# Metastatic melanoma patient outcomes since introduction of immune checkpoint inhibitors in England between 2014 and 2018

**DOI:** 10.1002/ijc.33266

**Published:** 2020-09-12

**Authors:** Ruth Board, Rebecca Smittenaar, Sarah Lawton, Hanhua Liu, Bukky Juwa, David Chao, Pippa Corrie

**Affiliations:** ^1^ Department of Oncology Lancashire Teaching Hospitals NHS Trust Preston UK; ^2^ National Cancer Registration and Analysis Service, Health Improvement Directorate Public Health England London UK; ^3^ Department of Oncology Royal Free London NHS Foundation Trust London UK; ^4^ Department of Oncology Cambridge University Hospitals NHS Foundation Trust Cambridge UK

**Keywords:** checkpoint inhibitors, metastatic melanoma, real world outcomes

## Abstract

Immune checkpoint inhibitors (CPIs) have radically changed outcomes for patients diagnosed with metastatic melanoma globally in the last 10 years, based on evidence of overall survival (OS) benefits generated from international randomised controlled trials (RCTs). Since RCTs do not always reflect real‐world prescribing, we interrogated established national databases to track prescribing of CPIs approved for first line treatment of metastatic melanoma patients in England since 2014 and determined patient outcomes associated with OS, as well as treatment‐related toxicity. Between April 2014 and March 2018, 5465 melanoma patients were diagnosed and treated with systemic anticancer therapy (SACT), 2322 of which received first‐line CPIs. There was good 3‐year OS concordance with RCT outcomes for ipilimumab (32%), ipinivo (56%) and nivolumab (51%), but OS was lower than expected for pembrolizumab (40%). Comparing patients prescribed ipinivo with those prescribed pembrolizumab, ipinivo‐treated patients were younger (88% vs 49% patients <70 years, *P* < .001) and fitter (60% vs 38% patients with Eastern Cooperative Oncology Group [ECOG] performance status 0, *P* < .0001). Emergency hospital admission rates from the earliest and last treatment dates were higher for patients prescribed ipinivo (37% and 55%) compared to those prescribed pembrolizumab (17% and 29%). The 30‐day mortality rates favoured ipinivo patients (3.8% ipinivo, 9.1% pembrolizumab, *P* < .0001) and likely reflected marked differences in median treatment durations: 63 (range 7‐440) days for ipinivo and 192 (range 5‐943) days for pembrolizumab. The dominant treatment‐related condition linked to hospital admission was colitis, recorded for 25% of patients prescribed ipinivo compared to 4% of patients prescribed pembrolizumab. Our population data has demonstrated that RCT outcomes can be achieved in routine care settings with careful patient selection.

## Abstract 2

**What's new?**

Immune checkpoint inhibitors (CPIs) greatly impact overall survival in metastatic melanoma. In England, CPIs have been used as first‐line therapy for this malignancy since 2014. This retrospective study assessed survival and toxicity among English metastatic melanoma patients prescribed CPIs between 2014 and 2018. For the CPIs ipilimumab, nivolumab, and ipinivo, survival outcomes were remarkably similar to registration trials. Poorer outcomes were associated with pembrolizumab, possibly because patients who received this drug were older and relatively less fit. Ipinivo generated the highest rates of emergency hospital visits and admissions, although 30‐day mortality was unchanged, potentially reflecting effective management of complications.

**Abbreviations**
CPI: checkpoint inhibitors
CTLA‐4: cytotoxic T‐lymphocyte‐associated protein 4
ECOG: Eastern Cooperative Oncology Group
HES: Hospital Episode Statistics
ICD10: International Statistical Classification of Disease 10th revision
Ipinivo: Ipilimumab and nivolumab
IrAE: immune‐related adverse event
NCRAS: National Cancer Registration and Analysis Service
NHS: National Health Service
NICE: National Institute for Health and Care Excellence
OS: overall survival
PD‐1: programme death‐1 receptor
PDS: Personal Demographics Service
PHE: Public Health England
PS: performance status
RCT: randomised controlled trials
SACT: systemic anticancer therapy
TA: technology appraisal

## 1 INTRODUCTION

Immune checkpoint inhibitors (CPIs) have markedly changed the treatment landscape for patients with metastatic melanoma over the last 10 years, due to proven impact on overall survival (OS) reported in international randomised controlled trials (RCTs).^1^, ^2^, ^3^, ^4^, ^5^ However, real‐world populations do not always reflect RCT populations controlled by strict trial eligibility criteria and poorer outcomes for patients treated outside clinical trial settings have been reported.^6^, ^7^ CPIs are high‐cost drugs with complex side effects that vary from very mild and manageable to life‐changing, as well as life‐threatening.^8^ Since the first reported OS benefits reported in patients diagnosed with metastatic melanoma, this new class of immunotherapy drugs is being used increasingly to treat a wide range of cancers and the resource implications for healthcare systems worldwide is concerning.^9^ The translation from trials to clinical practice in metastatic melanoma patient management provides a unique window through which to observe how CPIs were adopted across a national health service and have influenced population outcomes.

Since 2011, a series of CPI antibodies have been approved for the treatment of metastatic melanoma (encompassing unresectable locally advanced and distant metastatic spread). The CTLA‐4 antibody, ipilimumab, was the first systemic therapy to demonstrate OS benefit in this patient population in a RCT.^1^ Subsequently, the anti‐PD‐1 antibodies, nivolumab and pembrolizumab were shown to be more effective compared to either ipilimumab, or chemotherapy, respectively,^2^, ^3^ while also generating fewer severe, or life‐threatening side‐effects. The combination of ipilimumab and nivolumab (ipinivo) evaluated in the CheckMate 067 trial has the highest objective response rate and OS of all current first‐line metastatic melanoma immunotherapy options.^4^, ^5^ However, the OS gain associated with ipinivo compared to anti‐PD‐1 antibody alone was reported to be small (52% vs 44% at 5 years), while toxicity was far more severe (58% vs 23% severe or life‐threatening adverse events). There is currently no published national or international consensus guidance on selecting between anti‐PD1 monotherapy or ipinivo as first line treatment for patients with metastatic melanoma. However, the implications of treatment choice for both patients and healthcare systems can be profound, given that these antibodies are high cost drugs with complex and unpredictable side‐effects, administered for variable durations lasting months to several years in some instances.

In England, funding for, and hence access to, systemic anticancer therapy (SACT) within the National Health Service (NHS) is determined by the National Institute for Health and Care Excellence (NICE). Individual technology appraisals (NICE TAs) give guidance on new therapies with marketing authorisations based on their clinical efficacy and cost‐effectiveness.^10^ In the case of first‐line metastatic melanoma CPIs, all four regimens, ipilimumab, pembrolizumab, nivolumab and ipinivo received positive NICE guidance between July 2014 and July 2016, each within 1 to 8 months of their respective European marketing authorisations being granted. To better understand clinical practice, we reviewed the prescribing of first‐line CPIs for the treatment of patients with metastatic melanoma in England since their introduction in 2014 and used national databases to link outcomes including survival and treatment toxicity of treated patients.

## 2 MATERIALS AND METHODS

This work uses data that has been provided by patients and collected by the NHS as part of their care and support. The data is collated, maintained and quality assured by the National Cancer Registration and Analysis Service, which is part of Public Health England (PHE).

All individuals diagnosed with a melanoma skin cancer (ICD10 C43) in England between April 1, 2010 and December 31, 2017, were extracted from the National Cancer Registration Dataset.^11^ Cases were linked to the national SACT dataset^12^ using unique patient NHS numbers to identify those patients with confirmed unresectable locally advanced stage III or IV metastatic melanoma receiving first‐line CPI therapy with ipilimumab, pembrolizumab, nivolumab or ipinivo. First‐line therapy was confirmed by ensuring there was no record of any systemic therapy being prescribed for advanced disease prior to being prescribed a CPI.

The patient data was linked to the Hospital Episodes Statistics (HES)^13^ Inpatient and Accident and Emergency (A&E) datasets to establish how many patients either attended or were admitted within 30 days of their first and last CPI treatment record in the SACT database.^14^ A&E attendances correspond to when patients attend A&E. Emergency inpatient admissions are when patients are admitted for unplanned care. There are several routes through which a patient can be admitted for emergency care, including managed routes such as via a General Practitioner, as well as being admitted following an A&E attendance. Thirty‐day emergency hospital attendance or admission rates measured from first CPI treatment date were assessed, aiming to capture events more likely to be due to treatment‐related toxicity; 30‐day emergency hospital attendance or admission rates measured from the last CPI treatment date were expected to capture events more likely to be associated with treatment cessation, which could include disease progression, as well as treatment‐related toxicity. The conditions associated with emergency hospital admissions were identified by ICD‐10 codes recorded locally. These were collated and explored to determine whether and to what extent treatment‐related toxicities—in particular, immune‐related adverse events (IrAEs)—contributed to emergency hospital admissions. To examine for IrAEs of special interest, specific ICD‐10 codes (refer Table S1 for code names) were grouped to identify episodes of colitis (K521, K529 and A099), endocrinopathy (E059, E222, E230, E231, E236 and E871) and hepatitis (K711, K712, K716, K718, K754 and K759). In addition, we looked for any other frequently occurring ICD‐10 codes which accounted for >10% total recorded codes in any one treatment group.

### 2.1 Statistical analysis

OS was calculated using Kaplan‐Meier methodology from a patient's earliest CPI treatment date recorded in the SACT database and the patient's date of death, or the date the patient was last traced for whether they had died (vital status). All patients were traced for their vital status using the Personal Demographics Service (PDS)^15^ on October 2, 2019; this date was used as the censor date if patients were still alive. Patients were followed for a minimum of 18 months. To establish significant differences in OS, we compared 95% confidence interval estimates. Thirty‐day mortality rate was calculated from a patient's last treatment date recorded in the SACT database to their date of death.^14^ Thirty‐day emergency hospital attendance or admission rates were calculated from a patient's first and last treatment date recorded in the SACT database to any emergency attendance or admission dates recorded in the HES database. We used a two‐samples test of proportions to compare clinical characteristics and outcomes associated with patients prescribed ipinivo and patients prescribed pembrolizumab, or ipilimumab, and used the more conservative significance value of α = 0.01 to correct for these multiple statistical tests.

## 3 RESULTS

### 3.1 Patient characteristics and prescribing patterns

**FIGURE 1:**
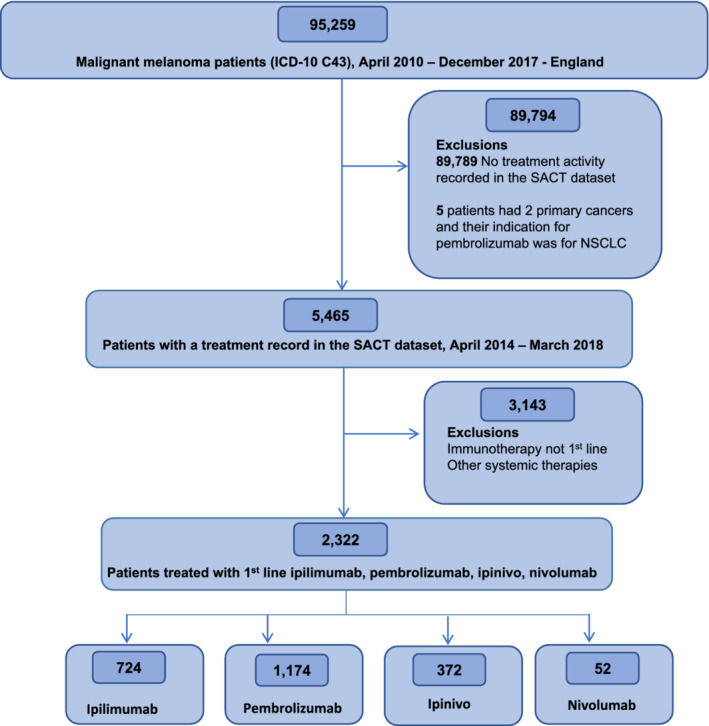
Consort diagram of patients diagnosed between April 2010 and December 2017 in England who received ipilimumab, nivolumab, ipinivo and pembrolizumab as first‐line treatment for metastatic melanoma [Color figure can be viewed at wileyonlinelibrary.com]

Between April 1, 2010 and December 31, 2017, 5465 patients were diagnosed with melanoma, of whom, 2322 received first‐line CPI treatment for metastatic disease within 56 different hospitals across England. The breakdown by treatment regimen was as follows: 724 patients were prescribed ipilimumab, 1174 were prescribed pembrolizumab, 52 were prescribed nivolumab and 372 were prescribed ipinivo (Figure 1).

**FIGURE 2:**
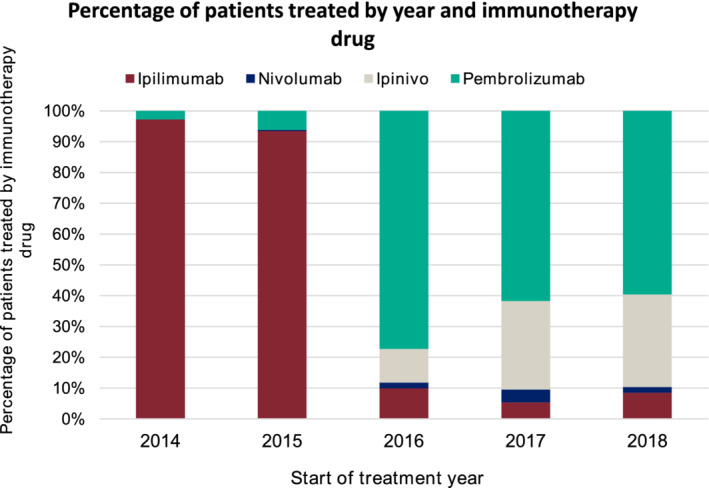
Annualised proportion of patients prescribed ipilimumab, pembrolizumab, nivolumab and ipinivo linked to patient access, determined by NICE TA guidance publications [Color figure can be viewed at wileyonlinelibrary.com]

As demonstrated in Figure 2 and Figure S1, prescribing of each regimen rapidly increased once access was granted by NICE. Prior to approval of anti‐PD‐L1 antibodies, ipilimumab accounted for nearly all CPI prescriptions in 2014 and 2015. In November 2015, following publication of clinical trial data confirming greater efficacy compared to ipilimumab, pembrolizumab was approved for use and, by 2016, was the dominant CPI prescribed. The choice of anti‐PD‐1 antibody continued to be predominantly pembrolizumab, despite access to nivolumab being granted in February 2016. From January 2016, prescribing of ipinivo increased annually and accounted for 1 in 3 CPI prescriptions by 2018.

Patients prescribed CPIs were predominantly adults spanning a wide age range; overall median age was 68 (mean 66, range 17‐97) years (Table 1). One patient prescribed ipinivo was under 18 years of age. Most patients were assessed as fit using the ECOG performance status (PS) scale of 0‐1, with less than 10% of patients being less fit (ECOG PS 2 or more). Comparing patients prescribed ipinivo and pembrolizumab, ipinivo‐treated patients were younger (81% vs 42% patients <70 years, *P* < .001) and fitter (60% vs 38% patients with ECOG performance status 0, *P* < .0001).

**TABLE 1:** Patient pretreatment characteristics

Patient characteristics	Ipilimumab (724)	Pembrolizumab (1174)	Nivolumab (52)	Ipinivo (372)
Sex				
Male	65%	63%	71%	66%
Female	35%	37%	29%	34%
Age				
<18	0%	0%	0%	0.3%
18‐29	1%	1%	4%	2%
30‐39	5%	2%	10%	7%
40‐49	11%	4%	10%	15%
50‐59	19%	14%	13%	26%
60‐69	31%	21%	27%	31%
70‐79	26%	34%	29%	18%
80+	7%	25%	8%	1%
Performance status				
0	43%	38%	48%	60%
1	25%	40%	27%	23%
2	3%	8%	6%	3%
3	0.3%	1%	2%	0%
Missing	29%	13%	17%	13%

The median (range) duration that patients remained on CPI treatment was 66 (range: 3‐147) days with ipilimumab, 63 (range: 7‐440) days with ipinivo, 191 (range: 5‐943) days with pembrolizumab and 364 (range: 27‐657) days with nivolumab (Table S2).

### 3.2 Efficacy outcomes

**FIGURE 3:**
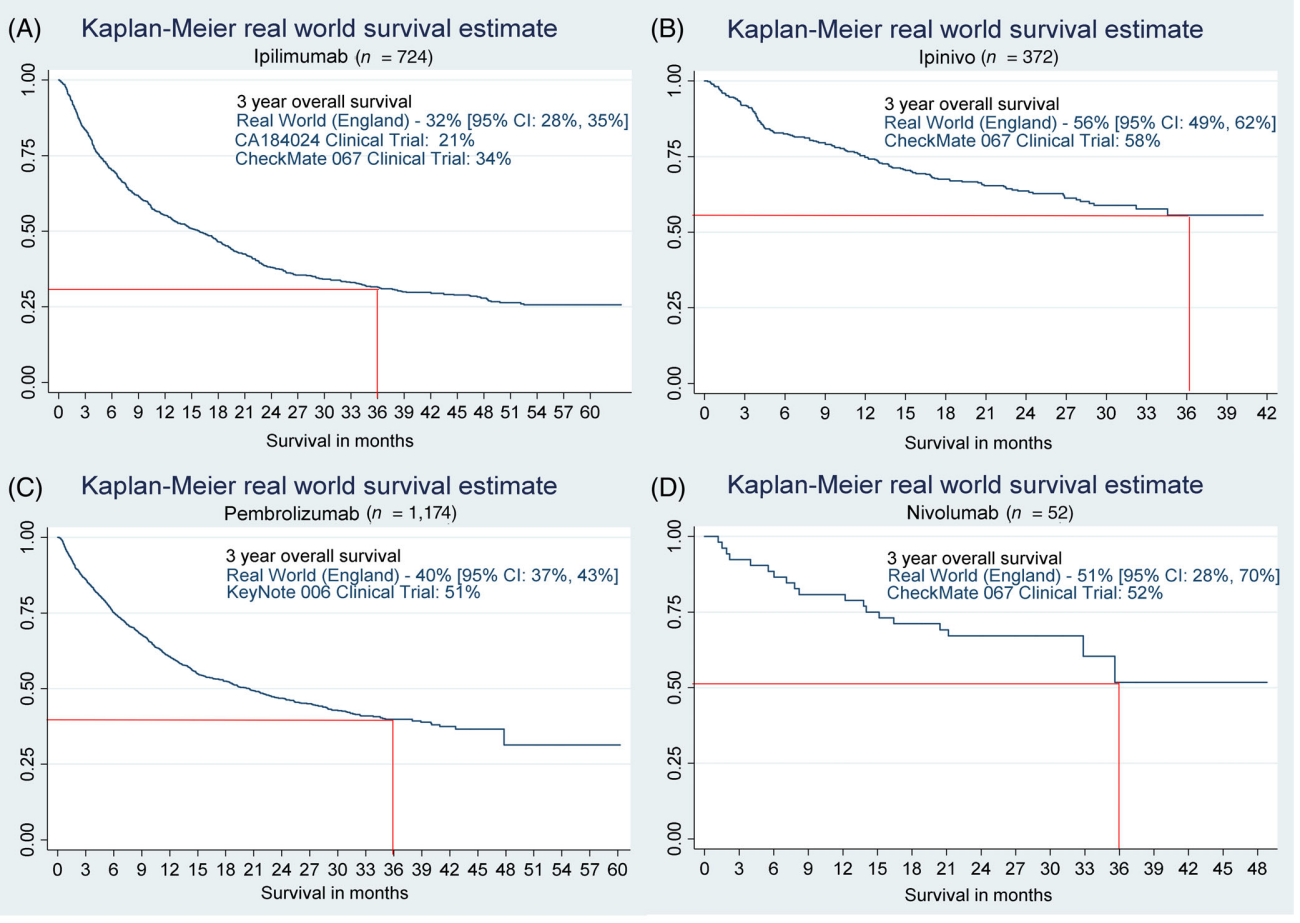
Overall survival of patients treated with, A, ipilimumab; B, ipinivo; C, pembrolizumab; and D, nivolumab [Color figure can be viewed at wileyonlinelibrary.com]

Three‐year OS associated with ipilimumab treatment was 32% [95% CI: 28%, 35%], 40% [95% CI: 37%, 43%] with pembrolizumab, 51% [95% CI: 28%, 70%] with nivolumab and 56% [95% CI: 49%, 62%] with ipinivo (Figure 3).

### 3.3 Toxicity outcomes

Thirty‐day emergency hospital attendance or admission rates measured from first CPI treatment date were assessed, aiming to capture events more likely to be due to treatment‐related toxicity. From patients’ first CPI treatment (Table 2), 30‐day rates of emergency admissions were higher in ipinivo (37%) compared to ipilimumab (23%) (although this difference was not significant following the multiple comparisons correction [*P* = .013]) and pembrolizumab (17%, *P* < .0001). Thirty‐day rates of A&E attendances from a patient's earliest CPI treatment were also significantly higher in association with ipinivo (16%) compared to ipilimumab (9%, *P* < .0001) or pembrolizumab (8%, *P* < .0001).

**TABLE 2:** Rates of Emergency Hospital Admissions and Attendances for patients receiving ipinivo, pembrolizumab and ipilimumab

	In‐patient emergency admissions		A & E attendance	
Within 30 days of first treatment date				
Ipinivo	37%		16%	
Pembrolizumab	17%	*P* < .0001	8%	*P* < .0001
Ipilimumab	24%	*P* = .013	9%	*P* < .0001
Within 30 days of last treatment date				
Ipinivo	55%		19%	
Pembrolizumab	29%	*P* < .0001	14%	*P* = .33
Ipilimumab	40%	*P* < .0001	15%	*P* = .33

*Note:* The *P*‐value is comparing the percentage of ipinivo emergency inpatient admissions and A&E attendances with the percentage of emergency inpatient admissions and A&E attendances for pembrolizumab and ipilimumab. A&E attendances correspond to when patients attend A&E. Emergency inpatient admissions are when patients are admitted for unplanned care. There are many routes through which a patient can be admitted for emergency care, including managed routes such as via a General Practitioner, as well as being admitted following an A&E attendance.

Thirty‐day A&E attendance or emergency admission rates measured from the last CPI treatment date were expected to capture events more likely to be associated with treatment cessation, which could include disease progression, as well as treatment‐related toxicity. Table 2 demonstrates emergency admissions were significantly higher with ipinivo (55%) compared to ipilimumab (40%, *P* < .0001), or with pembrolizumab (29%, *P* < .0001). Rates of 30‐day A&E attendances following a patient's last recorded CPI treatment were not significantly different in the three groups (19% with ipinivo, 14% with pembrolizumab and 15% with Ipilimumab, *P* = .33).

Thirty‐day mortality from a patient's last known CPI treatment was significantly lower in association with ipinivo (3.8%) compared to either pembrolizumab (9.1%, *P* < .0001), or ipilimumab (9.4%, *P* < .0001).

**FIGURE 4:**
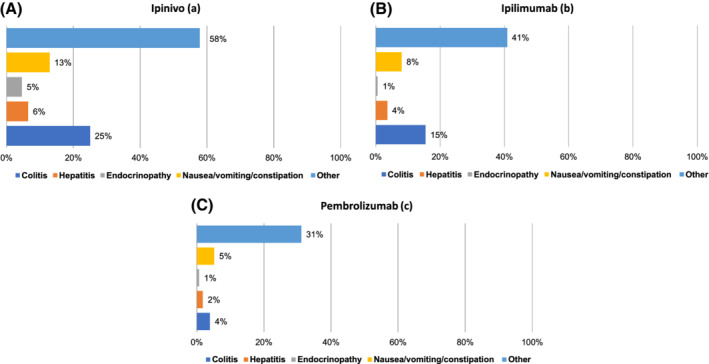
Percentage of patients having hospital admissions linked to selected ICD‐codes by treatment regimen: A, ipinivo; B, Ipilimumab; and C, pembrolizumab. Patients admitted with more than one complication will be counted multiple times [Color figure can be viewed at wileyonlinelibrary.com]

After excluding for ICD‐10 codes associated with melanoma diagnosis or co‐morbidities, a total of 922 ICD‐10 codes were linked to 889 unique patients admitted as an emergency. Only a small minority of these ICD‐10 codes could be strongly attributed to IrAEs (Figure 4). Colitis was the most frequent IrAE associated with hospital admission: 25% of ipinivo‐treated patients, 15% of ipilimumab‐treated patients and 4% of pembrolizumab‐treated patients had a hospital admission associated with colitis. ICD‐10 codes associated with hepatitis and endocrinopathies were recorded in 6% and 5% of ipinivo‐treated patients, 4% and 1% of ipilmumab‐treated patients and 2% and 1% of pembrolizumab‐treated patients. The combined ICD‐10 codes, R11X and K590, representing constitutional symptoms of nausea, vomiting and constipation, were the only other symptom set that occurred in >10% of patients, recorded in 13% of ipinivo‐treated patients, although were less often in ipilimumab‐treated (8%) and pembrolizumab‐treated (5%) patients.

## 4 DISCUSSION

This real‐world evaluation of first‐line CPI use in patients with metastatic melanoma in England demonstrates physician prescribing behaviour linked to drug access and the impact of selecting individual treatment regimens on key patient outcomes (survival and toxicity), as well as hospital resource use (reflected in emergency hospital attendances and admissions).

Ipilimumab was the first CPI introduced into clinical practice globally and there was rapid uptake in prescribing once national access to ipilimumab was granted in 2014. Pembrolizumab and nivolumab rapidly replaced ipilimumab as first‐line therapies by 2016, based upon randomised trials confirming superior efficacy as well as better tolerance compared to ipilimumab.^2^, ^3^ However, very little nivolumab was prescribed, which most likely reflected the priority given to dosing schedule, with a preference for the less resource‐intensive 3‐weekly infusion schedule associated with pembrolizumab compared to the 2‐weekly nivolumab infusion schedule being used at that time. Nine percent of first‐line CPI prescriptions in 2018 were for ipilimumab, which may represent a small cohort of patients who had relapsed on or after adjuvant anti‐PD‐1 antibodies being used in clinical trials around this time.

England was one of the first European countries to access ipinivo for routine use and prescriptions of this regimen increased annually following approval in 2016. By 2018, 1 in 3 patients were being prescribed ipinivo compared to single agent anti‐PD‐1 antibodies, reflecting rapid uptake of the combination CPI regimen reported to offer the highest OS compared to single‐agent CPI regimens in RCTs.^4^, ^5^

There was remarkably strong concordance between 3‐year OS outcomes in national clinical practice for patients prescribed ipilimumab, nivolumab and ipinivo compared to the respective RCTs. The exception was for those patients prescribed pembrolizumab, who had a lower than expected 3‐year OS of 40% compared to 51% reported in the KEYNOTE 006 trial.^17^ Access to prescribing most high‐cost drugs in England is limited to populations reflecting the registration trial entry criteria. However, while access to ipinivo is limited to patients with ECOG PS 0 or 1 with no active brain metastases (reflecting better prognostic groups), when pembrolizumab was first made available in England, access was not strictly limited in this way. The preponderance of older, less fit patients prescribed pembrolizumab may account for their poorer OS outcomes: entry into the KEYNOTE 006 trial^16^ was restricted to ECOG PS 0‐1 patients, while 9% of our real‐world pembrolizumab population had ECOG PS 2 or higher.

Selecting initial treatment with a single agent anti‐PD‐1 antibody or ipinivo is particularly challenging for melanoma specialists, since good predictive biomarkers are not available to help patients and clinicians weigh up the relatively modest gain in survival chances associated with ipinivo vs significantly increased risk of severe, potentially life‐threatening side‐effects.^5^ We identified 2‐fold higher rates of emergency hospital attendances and admissions within 30 days of starting ipinivo therapy compared to those occurring after starting pembrolizumab. This is despite the younger and fitter patient cohort prescribed ipinivo in comparison to pembrolizumab. The hospital admission rate after last treatment was also doubled in patients receiving ipinivo compared to pembrolizumab. Assessment of events occurring within 30 days of first treatment was anticipated to identify causes more likely to be treatment‐related, while events occurring within 30 days of last treatment was anticipated to identify causes more likely to be disease‐related.

The ipinivo regimen approved for treatment of metastatic melanoma is high dose ipilimumab (3 mg/kg) plus nivolumab (1 mg/kg) given on the same day once every 3 weeks for a total of 4 cycles, followed by maintenance nivolumab monotherapy; pembrolizumab is given intravenously on a 3‐weekly cycle; both regimens can be continued for as long as there is clinical benefit. The highest risk of toxicity events associated with ipinivo is during the first 12 weeks of combination therapy; median number of treatment cycles reported in the CheckMate 067 trial was 4, with 39% of patients discontinuing due to treatment‐related adverse events.^4^ In our population, the median duration of ipinivo treatment was 9 weeks, equivalent to receiving 3 out of 4 planned combination cycles. Therefore, it is reasonable to assume that events occurring after both first and last recorded ipinivo prescriptions could be grouped as treatment‐related events rather than disease‐related. The emergency hospital admission rates reported are therefore consistent with the high rate (59%) of severe and life‐threatening adverse events reported in the CheckMate 067 trial.^4^ In contrast, pembrolizumab is a much better tolerated drug, with severe and life‐threatening adverse events occurring in only 10% of patients treated in the KEYNOTE 006 trial^16^ and 7% discontinued due to treatment‐related toxicity. The most common reason for discontinuing anti‐PD‐1 monotherapy was disease progression. The higher rate of emergency hospital attendances and admissions within 30‐days of last pembrolizumab treatment compared to first pembrolizumab treatment as recorded in the SACT database might therefore be influenced by complications of uncontrolled metastatic melanoma.

As this was an observational rather than a randomised study, we cannot conclude rates of hospital admissions are a direct result of their CPI treatment or their metastatic melanoma rather than due to other different pre‐existing characteristics of the patient groups. However, the higher rates of colitis (25% vs 4%), hepatitis (6% vs 2%) and endocrinopathies (5% vs 1%) in patients receiving ipinivo compared to pembrolizumab mirror the higher rates of these IrAEs observed with the combination CPI regimen compared to single‐agent anti‐PD‐1 antibodies in clinical trials.^4^, ^5^ The incidence of these specific IrAEs recorded for our patient populations are in fact lower than those reported in the randomised trials. We have reported events associated with hospital admissions, while a proportion of patients experiencing these toxicities will be successfully managed in the clinic. Furthermore, reporting toxicities in real life populations is challenging. Review of the huge volume of hospital admission ICD‐10 codes identified that there were no “one size fits all” codes directly reflecting IrAEs. However, choice of codes was deliberately confined to those with high confidence that they reflected irAEs, so it is likely that the event rates reported here may be conservative and other reasons (such as nausea and vomiting, which were commonly reported) could equally well be linked to inflammation of the gastrointestinal tract, for example.

Reassuringly, there was a lower 30‐day mortality associated with ipinivo compared to other monotherapy regimens, potentially demonstrating effective management of treatment‐related toxicities in the hospital setting and the improved OS with this treatment. Our data therefore suggest that ipinivo is both a safe and effective treatment option in patients selected for their young age and good fitness.

On the other hand, we cannot use these data to make specific recommendations about selecting between CPIs to treat individual patients. There are inherent limitations when retrospectively analysing population databases. Data quality is not as stringent as prospective RCTs, and *BRAF* mutation status was not available for patients in the SACT database. The very small number of patients (n = 54) prescribed single agent nivolumab made analyses less reliable for this cohort. Nevertheless, NCRAS does undertake data quality checks on an ongoing basis focusing mainly on completeness of database fields and compliance. Furthermore, the large size of this population study as well as the overall similarities to clinical trial outcomes adds strength to our findings as being reliable and clinically relevant.

It is well recognised that patients recruited to RCTs are highly selected compared to patients treated in routine care.^6^, ^7^, ^17^, ^18^ Trial populations tend to be younger and fitter, so recommendations based on RCTs may not translate to real‐world populations. It is vital that population data is analysed to ensure trial outcomes are translated into benefit in real‐world settings and to monitor toxicities in patients not eligible for, or underrepresented in, prospective clinical trials. Our population‐based research findings can be used to complement rigorous RCT outcomes. This large scale, real‐world information about benefits and harms associated with CPIs as first‐line treatment of patients with metastatic melanoma provides strong reassurance that these complex drugs are both effective and safe with careful patient selection.

## CONFLICT OF INTEREST

R. B. has received payment for advisory boards and travel grants to attend meetings from MSD, BMS, Novartis and GSK. D. C. has received advisory board and speaker fees from BMS and MSD and funding from MSD for an investigator‐initiated study. P. C. has received advisory board and speaker fees from BMS and MSD. R. S., S. L., H. L. and B. J. have no conflicts of interest to declare.

5

## ETHICS STATEMENT

Public Health England collects data on the diagnosis and treatment of cancer patients as part of the care in the NHS under section 251 of the Health and Social Care Act (2006), without the requirement to seek consent from these patients. This legislation allows for the processing of this data for the purposes of surveillance and research. The study has obtained approval from the UK Patient Information Advisory Group (now the Confidentiality Advisory Group, under Section 251 of the National Health Service Act 2006 [PIAG 03(a)/2001]).

## Supporting information


**Table S1** Treatment duration (medians and ranges)
**Table S2** ICD‐10 code names
**Figure S1** Actual monthly prescriptions of (a) ipilimumab, (b) pembrolizumab, (c) nivolumab and (d) ipinivo recorded in the SACT database between April 2014 and 2018.

## Data Availability

Enquiries for accessing the National Cancer Registration and Analysis Service data can be made to the Office for Data Release (odr@phe.gov.uk).
