# Supplementary material for: Metastatic melanoma patient outcomes since introduction of immune checkpoint inhibitors in England between 2014 and 2018
Source: Int J Cancer. 2020 Sep 12;148(4):868–75. doi: 10.1002/ijc.33266 (PMC7821238; doi:10.1002/ijc.33266)

Metastatic melanoma patient outcomes since introduction of immune checkpoint inhibitors in England between 2014 and 2018

Ruth Board[1*], Rebecca Smittenaar[2*], Sarah Lawton[2], Hanhua Liu[2], Bukky Juwa[2], David Chao[3], Pippa Corrie[4]

Table of Contents

Supplementary Table 1: Treatment duration …………………………………………………..…1

Supplementary Table 2: ICD-10 code names……………………………………………..……1

Supplementary Figure 1: Actual monthly prescriptions of immune checkpoint inhibitors…2

Supplementary Table 1: Treatment durations (medians and ranges)

Immunotherapy regimen	No. patients	Median treatment duration	Min	Max
Ipilimumab	724	68 [95% CI: 62, 76]	3	147
Ipnivo	372	63 [95% CI: 62, 71]	7	440
Nivolumab*	52	364	27	657
Pembrolizumab	1,174	192 [95% CI: 172, 216]	5	943

*** Confidence intervals could not be produced as there were an insufficient number of events.**

Supplementary Table 2: ICD-10 code names

Toxicity Grouping	ICD codes	Code names
Colitis	K521	Toxic gastro-enteritis and colitis
	K529	Non-infective gastro-enteritis and colitis, unspecified
	A099	Gastroenteritis and colitis of unspecified origin
Endocrinopathy	E059	Thyrotoxicosis, unspecified
	E222	Syndrome of inappropriate secretion of antidiuretic hormone
	E230	Hypopituitarism
	E231	Drug-induced hypopituitarism
	E236	Other disorders of pituitary gland
	E871	Hypo-osmolality and hyponatraemia

Hepatitis	K711	Toxic liver disease with hepatic necrosis
	K712	Toxic liver disease with acute hepatitis
	K716	Toxic liver disease with hepatitis, not elsewhere classified
	K718	Toxic liver disease with other disorders of liver
	K754	Autoimmune hepatitis
	K759	Inflammatory liver disease, unspecified
Nausea, vomiting and constipation	R11X	Nausea and vomiting
	K590	Constipation

Supplementary Figure 1: Actual monthly prescriptions of (a) ipilimumab, (b) pembrolizumab, (c) nivolumab and (d) ipinivo recorded in the SACT database between April 2014 and 2018.

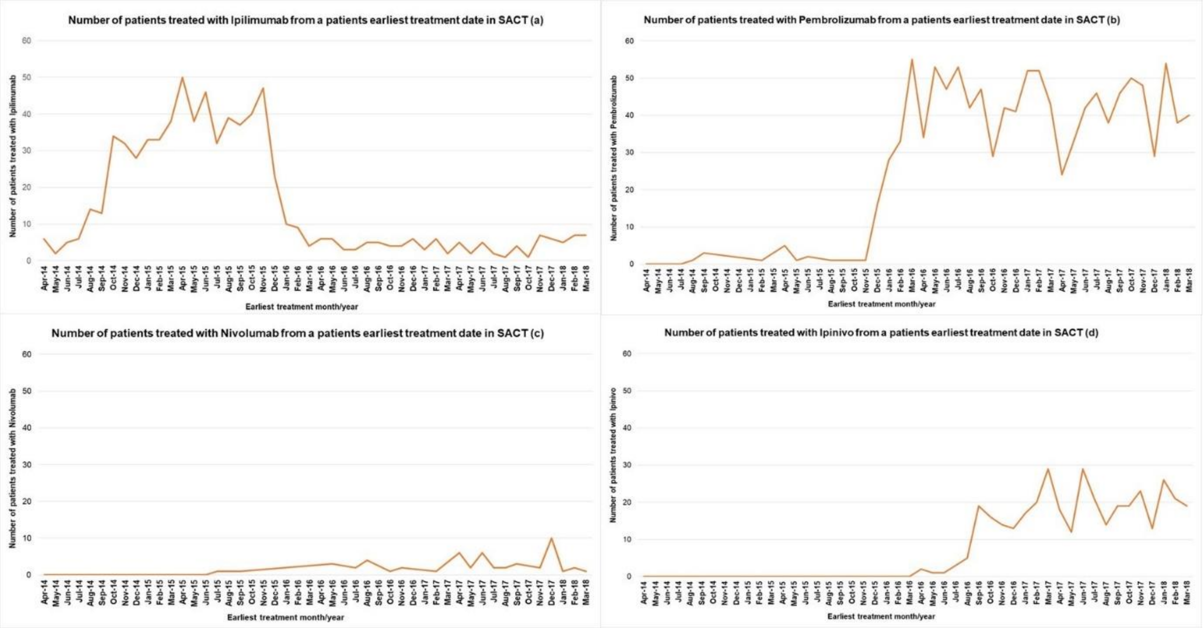

Supplement: Supplementary file 1 — Table S1 Treatment duration (medians and ranges) Table S2 ICD‐10 code names Figure S1 Actual monthly prescriptions of (a) ipilimumab, (b) pembrolizumab, (c) nivolumab and (d) ipinivo recorded in the SACT database between April 2014 and 2018. [file IJC-148-868-s001.pdf]
